# Le syndrome de Kallmann-de Morsier: à propos de trois cas

**DOI:** 10.11604/pamj.2019.33.221.11678

**Published:** 2019-07-18

**Authors:** Halima Marhari, Fatima Zahra Chahdi Ouazzani, Hanan El Ouahabi, Laila Bouguenouch

**Affiliations:** 1 Service d'Endocrinologie, Diabétologie et Nutrition, CHU Hassan II, Fès, Maroc; 2 Faculté de Médecine et de Pharmacie de Fès, Université Sidi Mohamed Ben Abdellah, Fès, Maroc; 3 Unité de Génétique Médicale et d'Oncogénétique, Laboratoire Centrale d'Analyses Médicales, CHU Hassan II, Fès, Maroc

**Keywords:** Hypogonadisme hypogonadotrope, mutation génétique, impubérisme, aménorrhée, anosmie, Hypogonadotrophic hypogonadism, genetic mutation, impuberism, amenorrhea, anosmia

## Abstract

Le syndrome de Kallmann-de Morsier (KS) est une maladie génétique du développement du système olfactif caractérisée par l'association d'un hypogonadisme hypogonadotrophique par déficit en gonadolibérine (GnRH) et d'une anosmie ou hyposmie. Le diagnostic se fait le plus souvent à l'adolescence devant une absence de puberté spontanée associée à un trouble de l'odorat, avec visualisation sur l'IRM hypothalamo-hypophysaire d'une hypoplasie voire une aplasie des bulbes et/ou des lobes olfactifs. Parfois, le diagnostic peut être évoqué dans la petite enfance devant l'association d'une cryptorchidie et d'un micropénis. Une mutation dans l'un de ses gènes connus n'est retrouvée que dans moins de 30% des cas et donc plusieurs autres gènes restent à découvrir. Grâce au traitement hormonal, la puberté se produit dans tous les cas, et la fertilité peut être obtenue dans la plupart des cas. Dans la présente étude, nous rapportons 3 cas de patients atteints de ce syndrome.

## Abstract (en)

Kallmann-de Morsier syndrome (KS) is a genetic disease of the olfactory system characterized by the association of hypogonadotropic hypogonadism also referred to as gonadotropin-releasing hormone (GnRH) deficiency and anosmia or hyposmia (with hypoplasia or aplasia of the olfactory bulbs). Apart from sporadic cases that occur most often, familial Kallmann’s syndrome is being described with increasing frequency. Diagnosis is mainly made in adolescents with absence of spontaneous puberty associated with smell disorders with hypoplasia or even aplasia of the bulbs and/or of the olfactory lobes on MRI. Sometimes, the diagnosis may be suspected in early childhood due to the association of cryptorchidism and micropénis. A mutation in one of known genes is only found in less than 30% of cases and, therefore, many other genes are still to be found. Hormone therapy allows pubertal growth in all cases and fertility can be obtained in most of the cases. We here report 3 cases of patients with this syndrome.

## Introduction

Le syndrome de Kallmann-de Morsier (KS) ou dysplasie olfacto-génitale, est défini par l'association d'un hypogonadisme hypogonadotrope et d'une anosmie ou hyposmie [1] et se caractérise par une hétérogénéité à la fois génétique et phénotypique. L'hypogonadisme est dû à des anomalies du développement neuronal affectant la migration prénatale des neurones à GnRH [2], alors que l'anosmie est secondaire à une atrophie des bulbes et/ou des lobes olfactifs. Cette maladie touche environ 1 garçon sur 10000 et est 4 fois moins fréquente chez la fille, il est possible que cette prévalence féminine soit sous-estimée du fait d'un diagnostic féminin moins visible. Le diagnostic se fait le plus souvent à l'adolescence devant une absence de puberté spontanée associée à un trouble de l'odorat, avec visualisation sur l'IRM hypothalamo-hypophysaire d'une hypoplasie voire une aplasie des bulbes et/ou des lobes olfactifs. Sur le plan génétique, deux formes sont décrites: la forme familiale et la forme sporadique qui reste la plus fréquente [3]. À ce jour, huit gènes ont été identifiés, néanmoins aucune mutation dans l'un de ses huit gènes n'est retrouvée chez environ 60 à 65% des patients. Sur le plan thérapeutique, le traitement hormonal a pour objectif de déclencher la puberté et de maintenir les caractères sexuels secondaires. Par les trois cas cliniques, suivis en ambulatoire dans le service d'Endocrinologie du CHU Hassan II de Fès, nous allons essayer de clarifier certains aspects clinico-biologiques, génétiques et radiologiques du KS chez une population marocaine.

## Patient et observation

**Figure 1:**
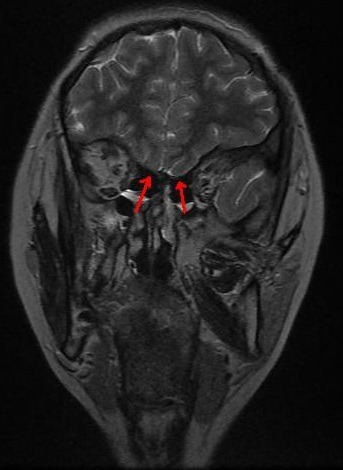
IRM cérébrale montrant une agénésie bilatérale des bulbes olfactifs

**Cas 1**: patient âgé de 20 ans, célibataire, sans antécédents pathologiques notables, qui présentait un impubérisme avec notion d'anosmie; chez qui l'examen clinique avait retrouvé un patient en surpoids, impubère avec des testicules intra-scrotaux de 1,5 cm x 1,5 cm en bilatéral, un micropénis (-2,5DS), une pilosité pubienne stade I de Tanner, une pilosité axillaire absente et une gynécomastie stade III. Au bilan biologique un hypogonadisme hypogonadotrope était noté: FSH=0.07mUI/ml (0,95-11,95) LH=0.08mUI/ml (1,14-8,75), testostéronémie basse à 0.5ng/ml, avec à l'IRM hypothalamo-hypophysaire une agénésie des bulbes olfactifs (Figure 1). L'étude génétique n'avait pas objectivé de mutations au niveau des gènes étudiés (KAL1, FGFR1, FGF8, CHD7, PROKR2, PROK2, HS6ST1, WDR11, SEMA3A, GNRH1, GNRHR, KISS1, KISS1R, SOX10, TAC3, TACR3), avec un caryotype 46 XY. Les autres axes hypothalamo-hypophysaires étaient indemnes et l'échographie rénale était sans particularité. Le patient était mis sous androgénothérapie (Enanthate de testostérone) avec une bonne évolution clinique: érection, éjaculation présentes, verge de 5cm. Pour sa gynécomastie le patient était adressé en chirurgie plastique pour une prise en charge esthétique.

**Cas 2**: patiente âgée de 22 ans, célibataire, sans antécédents pathologiques notables, qui présentait une aménorrhée primaire avec une anosmie chez qui l'examen clinique avait retrouvé une patiente maigre (IMC à 16,8 kg/m^2)^, impubère: les seins stade II, la pilosité pubienne stade III de Tanner et la pilosité axillaire peu fournie. Sur le plan biologique, un hypogonadisme hypogonadotrope était objectivé: FSH =0,74mUI/ml (2-10), LH= 0,09mUI/ml (0,5-5), œstradiol < 10pg/ml et sur le plan radiologique, l'IRM hypothalamo-hypophysaire avait montré une hypoplasie des bulbes olfactifs et une hypotrophie des gouttières olfactives. L'étude génétique était sans particularité notamment pas de mutations retrouvées avec un caryotype normal. L'exploration des autres axes hypothalamo-hypophysaires n'avait pas objectivé d'insuffisance et l'échographie rénale était normale. La patiente était mise sous traitement hormonale substitutif avec apparition des cycles menstruels réguliers.

**Cas 3**: patiente âgée de 30 ans, célibataire, sans antécédents pathologiques notables, qui présentait une aménorrhée secondaire sans anosmie ni hyposmie associée avec à l'examen clinique: patiente en obésité totale avec des caractères sexuels stade V de Tanner. Un hypogonadisme hypogonadotrope était noté: FSH=3,49mUI/ml (3-7), LH=1,55mUI/ml (0,5-5), Oestradiol<10pg/ml (140-400), Prolactine=21,58ng/ml, Testosterone=0,41ng/ml, HGPO: glycémie à T0=0,73g/l, à T120=0,75g/l (hyper-insulinisme) et à l'IRM hypothalamo-hypophysaire: une hypoplasie des bulbes olfactifs et hypotrophie des gouttières olfactives. L'étude génétique n'avait pas objectivé de mutations, l'exploration des autres axes hypothalamo-hypophysaires étaient sans particularité et l'échographie rénale n'avait pas objectivé de malformations ni d'anomalies. La patiente était mise sous traitement hormonale substitutif avec apparition des cycles menstruels réguliers. Pour le syndrome métabolique: la patiente était mise sous mesures hygiéno-diététiques associées à la metformine 1500mg/jr avec bonne évolution clinique et biologique.

## Discussion

Le syndrome de Kallmann a été décrit pour la première fois en 1944 par Franz Josef Kallmann, un généticien d'origine allemande émigré aux Etats-Unis; c'est une cause importante de défaut complet ou partiel, de développement pubertaire et résulte le plus souvent d'une sécrétion diminuée simultanée des 2 gonadotrophines hypophysaires, LH et FSH. Le tableau clinique du KS est variable suivant le sexe et l'importance du déficit. Il sera évoqué chez le garçon devant l'existence d'une cryptorchidie uni ou bilatérale et/ou d'un micropénis au cours de la période néonatale [4]. Le plus souvent, le diagnostic est suspecté devant une absence de développement pubertaire après l'âge de 14 ans, ce qui est le cas chez le patient rapporté dans la première observation. À ce stade, la présence d'un micropénis et/ou d'une cryptorchidie est très en faveur de l'hypogonadisme hypogonadotrope congénital et rend improbable le diagnostic de retard pubertaire simple qui est une cause beaucoup plus fréquente de retard pubertaire avec gonadotrophines basses [5]. Cependant la détection prépubertaire est facilitée quand il existe déjà un cas familial. Chez la fille, de même que notre deuxième cas rapporté, le KS est révélé par une aménorrhée primaire dans plus de 90% des cas; le développement mammaire est très variable, souvent présent et parfois presque normal [6]; concernant la pilosité pubienne, elle peut être absente, clairsemée ou même normale. Sous une forme atténuée, l'hypogonadisme peut être limité à une anovulation chronique, alors que la sécrétion d'œstradiol est adéquate pour le développement de l'endomètre laissant apparaitre une seule menstruation (aménorrhée primo-ordinaire) ou une oligoménorrhée chronique ou un test au progestatif positif [7]. Dans tous les cas, il faudra rechercher des signes cliniques associés fortement évocateurs du syndrome de Kallmann comme une anosmie, des syncinésies (mouvements en miroir), anomalie de l'attention visuelle, anomalie de la motricité oculaire, ptosis, syndrome cérébelleux, surdité, pieds creux, palais creux, fente labiale et/ou palatine, agénésie dentaire agénésie rénale.

Devant une suspicion clinique d'un SK, un bilan sanguin s'impose, qui va révéler un hypogonadisme hypogonadotrope (testostéronémie <3,5nmol/l chez le garçon et des concentrations sériques d'œstradiol faibles chez la fille, parfois en dessous de la limite de détection), avec des taux plasmatiques de LH et FSH bas ou paradoxalement normaux. La GnRH a une courte demi-vie, et ne peut être mesurée dans le sérum; cependant chaque pulse de GnRH est synchrone avec un pulse de LH, cette dernière est donc la mesure inférée de la GnRH. Pour l'inhibine B, on la retrouve diminuée par rapport au stade pubertaire [8]. L'évaluation des fonctions antéhypophysaires est impérative afin d'évaluer l'ensemble des fonctions hypophysaires; cette précaution permet de ne pas méconnaître d'autres insuffisances, tout particulièrement de l'axe corticotrope qui pourrait être peu parlante cliniquement. L'IRM s'est rapidement imposée comme examen de choix pour confirmer le diagnostic de syndrome de Kallmann de Morsier, en analysant les voies olfactives, situées au-dessus de la lame criblée de l'éthmoïde. Toutes les séquences permettent de déterminer l'absence ou non des bulbes olfactifs et l'épaisseur de la coupe ne doit pas excéder 3mm. Sa performance a été prouvée pour détecter de subtiles anomalies morphologiques notamment chez les enfants trop jeunes pour être testés du point de vue de l'olfaction [9] en montrant une agénésie ou une hypoplasie des bulbes olfactifs. Ainsi, il est impératif d'effectuer un examen uroradiologique détaillé chez les patients atteints de KS à la recherche des anomalies rénales en raison de leur fréquence et leur diversité [10].

Le KS est génétiquement hétérogènes et plusieurs modes de transmission génétique ont été décrits: récessif lié au chromosome X (KAL-1), autosomique dominant ou récessif; cependant, les cas sporadiques sont de loin les plus fréquents et seuls 30% environ des malades possèdent des mutations au niveau des gènes connus (KAL1, KAL2, PROK2, PROKR2, FGF8, FGF17, IL17RD, DUSP6, SPRY4, FLRT3, SEMA3A, GNRHR, CHD7, SOX10 et IL17RD) [11]. Alors, nos patients rapportés font partie de la population des 60% sans mutation génétique décelée. Pour le diagnostic moléculaire, il est important notamment chez les malades désireux de procréer afin d'évaluer, avant l'induction hormonale de la fertilité, le risque encouru par la descendance. Le traitement de l'hypogonadisme en cas de KS vise aussi bien de déclencher le développement pubertaire avec des injections de testostérone chez les hommes et des oestro-progestatifs chez les femmes puis assurer le maintien des caractères sexuels secondaires, que le développement de la fertilité, en faisant appel aux gonadotropines ou pulsatile GnRH pour obtenir la croissance testiculaire et la spermatogénèse chez les mâles ou l'ovulation chez les femelles, ce qui permet de restaurer la fécondité dans une grande majorité des cas [12]. Pour le traitement hormonal transitoire post natal des garçons atteints pour simuler la poussée postnatale des gonadotrophines, son efficacité n'est pas encore démontrée au terme de son impact ultérieur sur leur vie [13]. Concernant le conseil génétique, il doit être adapté à chaque famille et doit prendre en compte la forte variabilité phénotypique, y compris au sein d'une même famille.

## Conclusion

À la lumière de ce qui précède, le KS est une pathologie rare dont le diagnostic est basé sur les preuves cliniques de la maturation sexuelle arrêtée ou incomplète associées à une anosmie ou hyposomie (qui est un critère inconstant) et est confirmé par le dosage hormonal et l'IRM hypothalamo-hypophysaire centrée sur les bulbes olfactifs; toutefois l'étude génétique n'est pas toujours concluante vu que plusieurs gènes restent à découvrir, d'où l'intérêt de mener dans l'avenir des études génétiques plus approfondies dans ce sens. Ainsi, grâce au traitement hormonal, la puberté se produit dans tous les cas; à cet effet, un diagnostic précoce s'impose afin d'assurer une prise en charge optimale.

## Conflits d’intérêts

Les auteurs ne déclarent aucun conflit d'intérêts.
